# Longitudinal evaluation of T-cell responses to Pfizer-BioNTech and Janssen SARS-CoV-2 vaccines as boosters in Ghanaian adults

**DOI:** 10.3389/fimmu.2025.1643083

**Published:** 2025-09-12

**Authors:** Frank Osei, Kekeli Korshi Tudzi, Isaac Otieno Othol, Selorm Philip Segbefia, Diana Ahu Prah, Evans Nii Armah-Vedjesu, Abigail Naa Adjorkor Pobee, Oscar Nii Otto Darko, Theophilus Brenko, Doreen Teye-Adjei, Stella Nartey, Jones Amo Amponsah, Vincent Amarh, Godfred Futagbi, Dorcas Obiri-Yeboah, Frederica Dedo Partey, Michael Fokuo Ofori, Kwadwo Asamoah Kusi

**Affiliations:** ^1^ Department of Immunology, Noguchi Memorial Institute for Medical Research, College of Health Sciences, University of Ghana, Accra, Ghana; ^2^ Department of Medical Biochemistry, University of Ghana Medical School, College of Health Sciences, University of Ghana, Accra, Ghana; ^3^ Department of Animal Biology and Conservation Science, College of Basic and Applied Sciences, University of Ghana, Accra, Ghana; ^4^ Department of Microbiology and Immunology, School of Medical Sciences, University of Cape Coast, Cape Coast, Ghana; ^5^ West African Centre for Cell Biology of Infectious Pathogens, College of Basic and Applied Sciences, University of Ghana, Accra, Ghana

**Keywords:** COVID-19, SARS-CoV-2, cytokines, vaccines, booster shot, immune response, Ghana

## Abstract

**Introduction:**

In Ghana, at least five different COVID-19 vaccines based on mRNA or adenovirus vector delivery platforms have been authorized by the Ghana Health Service for vaccination. Although these vaccines have been instrumental in the control of COVID-19, data on the longevity of induced immunity in vaccinated individuals in Ghana is limited. This study aimed at assessing the cellular immune response kinetics among Ghanaians receiving booster vaccinations with the mRNA-based Pfizer and adenovirus-based Janssen COVID-19 vaccines.

**Methods:**

We conducted a longitudinal study using 48 Ghanaian adults who had completed primary vaccination series and administered a booster shot with either of the two vaccines. Pre-booster blood samples were collected to serve as the baseline, and post-booster samples at months 3, 6, and 9 for immunological analysis. T-cell responses were assessed using Luminex multiplex assay following stimulation of Peripheral Blood Mononuclear Cells (PBMCs) from study participants with SARS-CoV-2 antigens, whereas immune checkpoint molecules expression was assessed by flow cytometry.

**Results:**

Appreciable levels of the Th1 cytokines IL-1β, IL-6, IFN-γ and TNF-α and low levels of IL-2, IL-12 and IL-17A were observed in both groups. The Janssen vaccine booster elicited a more sustained cellular response over the nine months, while the Pfizer vaccine booster group showed signs of response decline after three months. Further sub-analysis showed that persons who received an mRNA-based primary vaccination before a viral vector vaccine booster had more durable cytokine responses. Checkpoint molecules, PD-1, CTLA-4 and TIM-3 were expressed at low levels (<10% of CD4+ or CD8+ T cell population with p-values > 0.05) and comparable between the two groups over the nine months.

**Discussion/conclusions:**

Levels of some cytokines were generally more sustained in the Janssen group compared to the Pfizer group. Heterologous vaccine recipients exhibited more efficient cellular immune responses compared to homologous recipients. In addition, T-cell inhibitory molecule kinetics suggests an efficient T-cell activity. These findings may have implications for the overall induction of long-term protective immunity by the two vaccine types.

## Introduction

Severe Acute Respiratory Syndrome Coronavirus 2 (SARS-CoV-2) is responsible for the COVID-19 pandemic which began in December 2019. As of June 2024, with over 775 million confirmed cases and 7 million deaths worldwide, it is the deadliest pandemic of the 21^st^ century (1). COVID-19 causes asymptomatic infections or mild symptoms such as chills, fever, myalgia, taste and smell loss, coughing, and exhaustion, with severe cases leading to conditions such as pneumonia, acute respiratory distress syndrome, cardiomyopathy, and encephalopathy (2, 3). SARS-CoV-2 is transmitted either directly through respiratory droplets and aerosols or indirectly through contaminated surfaces. In Africa, the epidemiology of COVID-19 was unique, with fewer mortality and morbidity reported and over a third of reported cases being asymptomatic (4–6). SARS-CoV-2 is a single-stranded, positive-sense, non-segmented RNA virus with an envelope, and belongs to the beta-coronaviruses genus of the *Coronaviridae* family with its genetic sequence closely resembling that of SARS-CoV-1 and MERS-CoV (7–9).

Immune protection against SARS-CoV-2 is achieved by natural infection and vaccination. Among the various preventive measures, the development of vaccines against SARS-CoV-2 was unparalleled, involving several novel platforms. These included Janssen (Ad26.COV2. S), Pfizer (BNT162b2), Oxford-AstraZeneca, Moderna (mRNA-1273), and Sputnik V vaccines, all of which encode the full-length Spike protein of SARS-CoV-2 (10–13). SARS-CoV-2 vaccination initiates CD4+ T-cell response, beginning with the activation of naïve CD4+ T cells and their differentiation into effector T cells, which direct antiviral function (14). Effector T-cells trigger the production of cytokines that coordinate activation, maturation, and function of T and B lymphocytes to generate antigen-specific immune responses to vaccination or natural infection. After T-cells complete their effector role, many of them die off, with a few remaining as long-lived cells. A fraction of T cells also differentiates into memory cells, which may persist for years even after viral clearance (15). Also, COVID-19 is characterized by an excessive inflammatory reaction marked by increased levels of various pro-inflammatory cytokines, including IL-1β, IL-6, TNF-α, IL-12, IFN-γ, IL-17 and others (16).

Based on statistics available to the Ghana Health Service, as of December 2023, over 28 million COVID-19 vaccine doses had been administered to 14.87 million persons (43.7% of the 34 million population) in Ghana, with 11.78 million persons (34.6%) completing the required vaccination schedule. The vaccines administered included Pfizer, Moderna (mRNA-based vaccines) as well as AstraZeneca, Janssen and Sputnik-V which used non-replicating viral vector platforms, each with distinct immunological profiles. mRNA vaccines have been shown to be safe and elicit higher levels of neutralizing antibody titers and T-cell responses; however, these wane more rapidly than the responses elicited by viral-vectored vaccines, which induce more robust and longer-lasting T-cell and antibody responses (12, 17). The observed waning immunity over time, coupled with the emergence of SARS-CoV-2 variants exhibiting substantial Spike protein divergence from the original strain used for vaccine development, indicates a potential decrease in vaccine efficacy against infection and/or disease (18, 19). This waning immunity could be complemented with intermittent booster vaccinations which have been partly shown to overcome T-cell exhaustion, although the duration is not clearly defined. Contrarily, prolonged vaccine-induced T-cell stimulation can lead to an immunological imbalance (20). These phenomena necessitate the development of new vaccines that account for spike protein diversity or the use of repeated booster shots of existing vaccines to ensure the maintenance of clinically relevant levels of immunity against COVID-19.

With African countries facing the challenge of access to COVID-19 vaccines during the pandemic, different combinations of vaccines were sometimes administered to recipients of vaccines that required two shots to complete vaccination (21, 22). In this study, we sought to assess the immunological outcomes of booster vaccinations administered in either a homologous (booster vaccines developed on the same or similar platforms to vaccines with which participants were previously vaccinated) or heterologous (booster vaccines developed on different platforms to vaccines with which participants were previously vaccinated) regimen. By examining these regimens, we seek to better understand the magnitude and quality of immune responses in individuals receiving either type of booster. Emerging evidence suggests that heterologous booster vaccination may enhance immune responses by engaging different antigen-presenting cells, stimulating diverse T- and B-cell responses, ultimately leading to broader and more durable cellular and humoral immunity (23). Previous studies have shown that heterologous boosting can enhance both humoral and cellular immune responses, offering a prospective benefit of broader and more durable immunity against variants of SARS-CoV-2 (24, 25).

While SARS-CoV-2 vaccination outcomes have been extensively researched, there is much less information on the kinetics of the cellular immune response, as most studies have focused on humoral immunity. This study investigated cellular immune responses to the Pfizer and Janssen COVID-19 vaccines over a nine-month period after their use as boosters in a cohort of fully vaccinated Ghanaians. By analyzing T cell frequencies, cytokine production, and immune checkpoint molecule expression, we aimed to elucidate the dynamics of cellular immunity, evaluate the effects of different vaccine combinations, and identify the optimal timing for periodic booster vaccination. The findings from this study are vital for evidence-based recommendations for COVID-19 booster strategies and provide critical insights for optimizing vaccination approaches to achieve long-term immune protection. We hypothesized that Janssen booster recipients will exhibit a more durable immune profile compared with Pfizer over a nine-month period. Additionally, heterologous vaccination would induce more durable cellular responses than homologous regimens.

## Materials and methods

### Study design, location and sample size

This study was conducted in Legon, an urban suburb of Accra, the capital of Ghana and its surrounding communities from November 2022 to October 2023. This was part of a larger longitudinal study titled “Comparative Assessment of Immunological Response and Response Longevity of Different COVID-19 Vaccines within the Ghanaian Population” based at the Noguchi Memorial Institute for Medical Research (NMIMR), University of Ghana. Study participants had completed the primary vaccination series (either a single or double dose regimen) at least 6 months prior to the start of this study and were eligible to receive a booster dose. Participants were enrolled at vaccination centers in and around the University of Ghana– Legon campus. Venous blood samples were drawn before booster vaccination and subsequently at months 3, 6 and 9 after booster vaccine administration for immunological analysis. For this study, samples from forty-eight (48) persons, including 24 who received the Janssen vaccine booster and 24 who received the Pfizer vaccine booster, were used. The sample size of 48 is based on sample size estimated in G*Power software (version 3.1.9.7) to be able to detect a medium effect size of 0.25 between the two booster vaccination groups with a power of 0.8 and at an alpha level of 0.05.

### Ethical considerations

Ethical approval was sought from the Institutional Review Board of NMIMR (NMIMR-IRB, approval number CPN 010/22-23), after obtaining scientific approval from the NMIMR Scientific and Technical Committee. Written informed consent was sought from each study participant prior to inclusion in the study. All experiments were conducted in compliance with the principles of the Belmont Report and the guidelines of the Declaration of Helsinki.

### Sampling procedure

Forty-eight (48) persons who had completed a primary COVID-19 vaccination regimen and met our inclusion criteria were recruited into the study. The inclusion criteria were persons aged between 18–70 years with a hemoglobin concentration of 10 g/dl or more for females and 12g/dl for males and a negative pregnancy test for females. Forty milliliters (40 ml) of venous blood were collected from each participant into heparin tubes before the booster vaccine administration. At each of months 3, 6 and 9 after booster vaccine administration, 40 ml of venous blood were again collected. Collected samples were transported to the laboratories of the Immunology Department at NMIMR for peripheral blood mononuclear cells (PBMCs) isolation.

### Peripheral blood mononuclear cell isolation, storage and retrieval

PBMCs from heparinized blood samples were isolated using the density gradient centrifugation method with Ficoll-Paque™ PLUS (Cytiva, Sweden). Twenty (20) ml of whole blood collected from participants were added to 20 ml of R0 (RPMI-1640 with L-glutamine and penicillin-streptomycin) to achieve a two-fold dilution. Twenty-five (25) ml of diluted blood was gently overlayed on 15 ml of Ficoll-Paque™ PLUS in a 50 ml falcon tube and centrifuged at 1200 x g for 20 minutes at room temperature without brakes. The middle ring band of mononuclear cells were collected using a 10 ml serological pipette into a new tube and topped up to 40 ml with cell wash medium (5% Fetal Bovine Serum in RPMI-1640). This was then centrifuged at 300 x g for 10 minutes at room temperature without brakes and the supernatant aspirated and discarded. After loosening cells, 30 ml of cell wash medium were added and centrifuged at 300 x g for 10 minutes at room temperature without brakes. Supernatants were discarded and isolated cell pellets were resuspended in 10 ml of cell wash medium, prior to counting and cryopreservation. Ten (10) µl of cell suspension was added to 10 µl of 0.4% Trypan blue for cell viability and concentration estimation using an automated cell counter. Cells were stored at 10 x10^6^ or 20x10^6^ cells per vial in 90% Fetal Bovine Serum + 10% Dimethyl Sulphoxide (Life Sciences Technology, UK) first in strata coolers at -80°C and then transferred to liquid nitrogen after 24 hours. Before experiments, stored PBMCs were thawed at 37°C in a water bath for 1 minute and subsequently washed with R10 (10% FBS, 1% Penicillin-Streptomycin in a specific volume of RPMI). The cells were then counted, rested for 1 hour in a water-jacketed incubator at 37°C and an atmosphere of 5% CO_2_, and counted just after the 1-hour rest.

### SARS-CoV-2 Spike receptor binding domain RBD protein expression

The receptor binding domain (RBD) of the Spike (S) protein of the SARS-CoV-2 ancestral strain was recombinantly expressed in 293 freestyle cell systems and purified to homogeneity as described by Wrapp et al. (26).

### PBMCs Stimulation with phytohemagglutinin and S-RBD protein of SARS-CoV-2

Rested cells were stimulated with PHA (2.5 µg/ml) as a positive control, as well as with RBD antigens (10 µg/ml) at a total volume of 500 µl per well using 24 well culture plate in a water-jacketed incubator at 37°C and 5% CO_2_ for 48 hours. After the incubation, supernatants (200 µl) were harvested into 96-well culture plates (Corning Incorporated, USA) and stored at -80 °C for Luminex Multiplex assay. The remaining cell fraction was transferred to FACS tubes and stained for flow cytometry, as described below.

### Flow cytometric detection of immune markers

Cells in FACS tubes were washed with 1X PBS and Staining buffer. They were subsequently stained with fluorescent-labeled CD3 (PerCP-Cy5.5; clone: UCHT1), CD4 (BV421; clone: SK3), CD8 (BV650; RPA-T8), CD279 (PE-Cy7; clone: EH12.1), CD152 (APC; clone: BNI3), CD45RO (FITC; clone: UCHL1) and CD366 (PE; clone:7D3) mouse anti-Human antibodies from BD Biosciences. The stained cells were incubated in a dark environment for 30 minutes and then washed with staining buffer. Fluorescence light scatter of cell populations was acquired using the BD LSR Fortessa X- 20 (BD Biosciences, USA). (See **Supplementary Figure 1** for gating strategy).

### Luminex multiplex assay

The Luminex multiplex assay was used to evaluate the levels of nine cytokines which are key in the induction of T-helper 1, T-helper 2, T-helper 17 and immunoregulatory responses and in COVID-19. The Human Premixed Multi-Analyte kit (Invitrogen, USA; 9-plex panel with TNF-α, IL-1β, IL-12p70, IL-17A, IL-2, IL-4, IFN-γ, IL- 10, and IL-6) was used according to the manufacturer’s instructions (Invitrogen, USA). Fifty microlitres (50 µl) of R10 was added to the lyophilized antigen standard, vortexed for 10 seconds and incubated on ice for 10 minutes to ensure complete reconstitution. Twenty-five microlitres (25 µl) of the reconstituted standards were added to 225 µl of R10, followed by four-fold dilution according to the manufacturer’s instruction. After standard preparation, 50 µl of vortexed magnetic bead solution were added to each well of the 96-well Luminex plate. The plate was washed three times with 150 µl of 1X wash buffer. Fifty microliters (50 µl) of prepared standard, blank (R10), and culture supernatants (samples) were added to their designated wells and kept on a plate shaker for 30 minutes at a speed of 500 x g at room temperature, after which it was incubated overnight at 4°C. After overnight incubation, the plates were placed on a plate shaker for 30 minutes at a speed of 500 x g, followed by washing three times with 150 µl per well of wash buffer. Twenty-five microliters (25 µl) of detection antibody were added to each well as the next step, incubated on a plate shaker for 30 minutes at a speed of 500 x g, and then washed three times. Fifty microliters (50 µl) of Streptavidin-PE were added to each well, and the plate was incubated for 30 minutes on a shaker at 500 x g and washed three times. After this step, 50 µl of amplification reagents 1 and 2 were added to each well, followed by incubation for 30 minutes on a shaker at 500 x g and washed three times. Finally, 120 µl of reading buffer was pipetted into each well and incubated for 5 minutes on a shaker at 500 x g. The plates were then read using the Luminex MAGPIX analyzer (XMAP Technology, USA).

### Data and statistical analyses

Comma-Separated Values (CSV) files containing the mean fluorescence intensity (MFI) data were obtained from the MAGPIX analyzer and uploaded onto the kit manufacturer’s online analysis app (ProcartaPlex app, ThermoFisher scientific). The app converts MFI data into analyte concentrations (pg/ml) using the titrated standards for each analyte contained in the multiplex panel. Flow cytometry files (FCS) were exported from the BD LSR Fortessa X- 20 and uploaded into the FlowJo analysis software (version 10.10) and the appropriate gating to assess expression levels of the selected immune markers (%) performed.

Friedman’s test was used to assess the longevity of the cellular response and immune checkpoint molecule expression across the four sampling timepoints, followed by Dunn’s multiple comparison *post-hoc* test where necessary. The magnitude of the induced response between the Janssen and Pfizer COVID-19 vaccine recipients at each of the study timepoints were also assessed using the Mann-Whitney U-test. GraphPad Prism (version 9.0) was used for both statistical analysis and graphical presentations. A generalized linear mixed effects model was fitted to the data in the R statistics environment using the lme4 package and the glmer function. The GLMM analysis estimates the fixed effect (booster type) on cytokine levels, while adjusting for covariates (age, sex and prior vaccine type), taking into account random error variance due to differences between participants and the different sample timepoints. The model equation used: [cytokine_high ~ booster*prior_vaccine + age + sex + time + (1 | subject_id)]. Cytokine levels were dichotomized as ‘high’ or ‘low’ based on a mean+2 S.D split, with values above being classified as high and those at or below classified as low. A p-value of ≤ 0.05 was considered statistically significant.

## Results

### Demographic characteristics of study participants

Samples from 48 participants were used in this study, with 24 participants receiving the Pfizer booster vaccine and the other 24 receiving the Janssen booster vaccine. Both groups consisted of 16 males and eight females. The median age of both the Janssen and Pfizer booster recipients was 26 years. Twenty-nine (29) participants received booster vaccines developed on similar delivery platforms as their primary vaccines (homologous group), while 19 received boosters developed on a different platform from their primary vaccines (heterologous group). The participants’ ages, sex, and primary vaccination statuses were comparable (**Table 1**).

**Table 1:** Demographic characteristics of study participants.

Variable	Level	Booster vaccines		p-value
		Janssen (n=24)	Pfizer (n=24)	
Age	mean (S.D)	26.0 (10)	26.0 (9.6)	0.63
Sex	males, n (%)	16 (66.67)	16 (66.67)	>0.99
	females, n (%)	8 (33.33)	8 (33.33)	
Prior vaccination platform	Viral vector, n (%)	17 (70.83)	12 (50)	0.24
	mRNA, n (%)	7 (29.17)	12 (50%)	

Comparison between groups were performed using the Fisher’s test for categorical variables and the Mann Whitney U T test for age. Statistical significance was defined as P ≤ 0.05 was considered statistically significant, SD: Standard deviation

### Individuals who received either the Janssen or Pfizer booster vaccines over nine months elicited appreciable levels of cytokine responses

**Figure 1:**
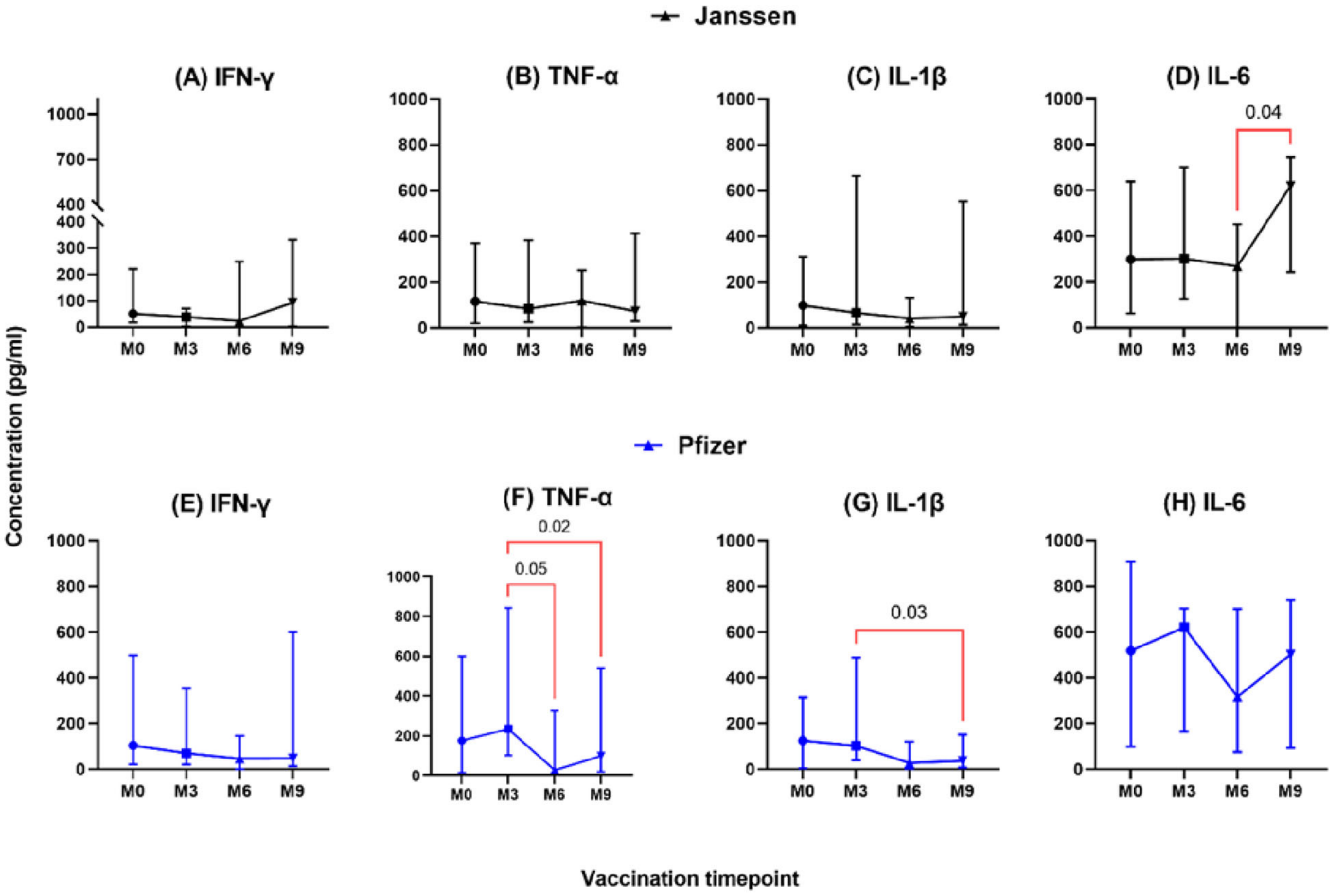
Longitudinal analysis of IFN-γ, TNF-α, IL-1β & IL-6 responses (pg/ml) in individuals who received the Janssen **(A–D)** or Pfizer **(E–H)** booster vaccines. Friedman’s test and Dunn’s multiple comparisons were used for across timepoints comparison within the Janssen and Pfizer groups. M0 = Samples taken before booster administration; M3, M6, and M9 = Samples taken at 3, 6, and 9 months after booster vaccine administration. Data points represent the median and the error bars, the minimum to maximum range.

The longevity of booster-induced cytokine responses was assessed and categorized as T helper 1 (IL-1β, IFN-γ, TNF-α, IL-2, IL-6, and IL-12), T helper 2 (IL-4), T helper 17 (IL-17), and immunoregulatory (IL-10)-associated cytokines. In both Janssen and Pfizer booster groups, comparable levels of T helper-1 associated-cytokines IL-1β, IFN-γ, TNF-α, and IL-6 were expressed at pre- and post-booster time points with no statistically significant differences observed (**Figure 1**). In the Janssen booster group, IL-6 levels were significantly higher at month 9 (Median: 620 pg/ml, [IQR: 154-746]) compared to month 6 (Median: 268 pg/ml; [IQR: 0.10-596], p=0.04, **Figure 1D**), whereas in the Pfizer booster group, TNF-α and IL-1β levels were significantly lower at month 9 (99 pg/ml [14-570]; 37[7-276]) compared to month 3 (235 pg/ml[46-854], p=0.02; 103 pg/ml[22-492] p=0.03; **Figures 1F, G**). TNF-α levels also declined significantly at month 6 (28 pg/ml [8-377]) compared to month 3 (235 pg/ml [46-854]; p=0.05, **Figure 1F**).

**Figure 2:**
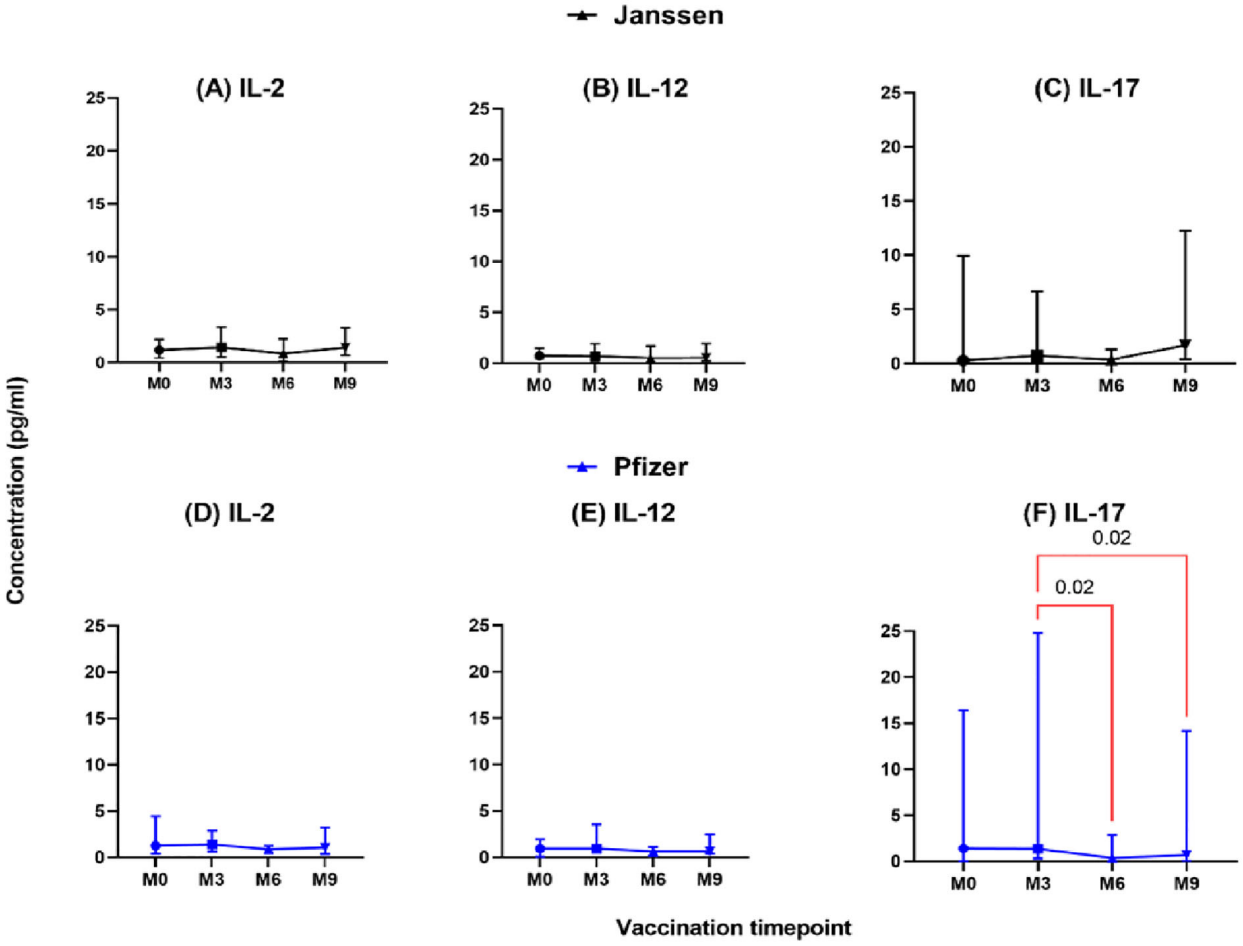
Longitudinal analysis of IL-2, IL-12, and IL-17 responses (pg/ml) in individuals who received Janssen **(A–C)** or Pfizer **(D–F)** booster vaccines. Friedman’s test and Dunn’s multiple comparisons were used for comparisons across time points in the Janssen and Pfizer recipients. M0 = Samples taken before booster administration; M3, M6, and M9 = Samples taken at 3, 6, and 9 months after booster administration. Data points represent the median and the error bars, the minimum to maximum range.

In the Janssen booster vaccine group, low levels of IL-2, IL-12, and IL-17 were expressed, and this was not significantly different pre- and post-booster vaccination (**Figures 2A–C**). A similar trend was seen in the Pfizer vaccine recipients for IL-2 and IL-12 (**Figures 2D, E**); however, IL-17 levels were significantly higher at month 3 (1.4 pg/ml [0.32-37]) compared to month 6 (0.37 pg/ml [0.02-3.3], p=0.02) and month 9 (0.70 pg/ml [0.03-18], p=0.02, **Figure 2F**).

**Figure 3:**
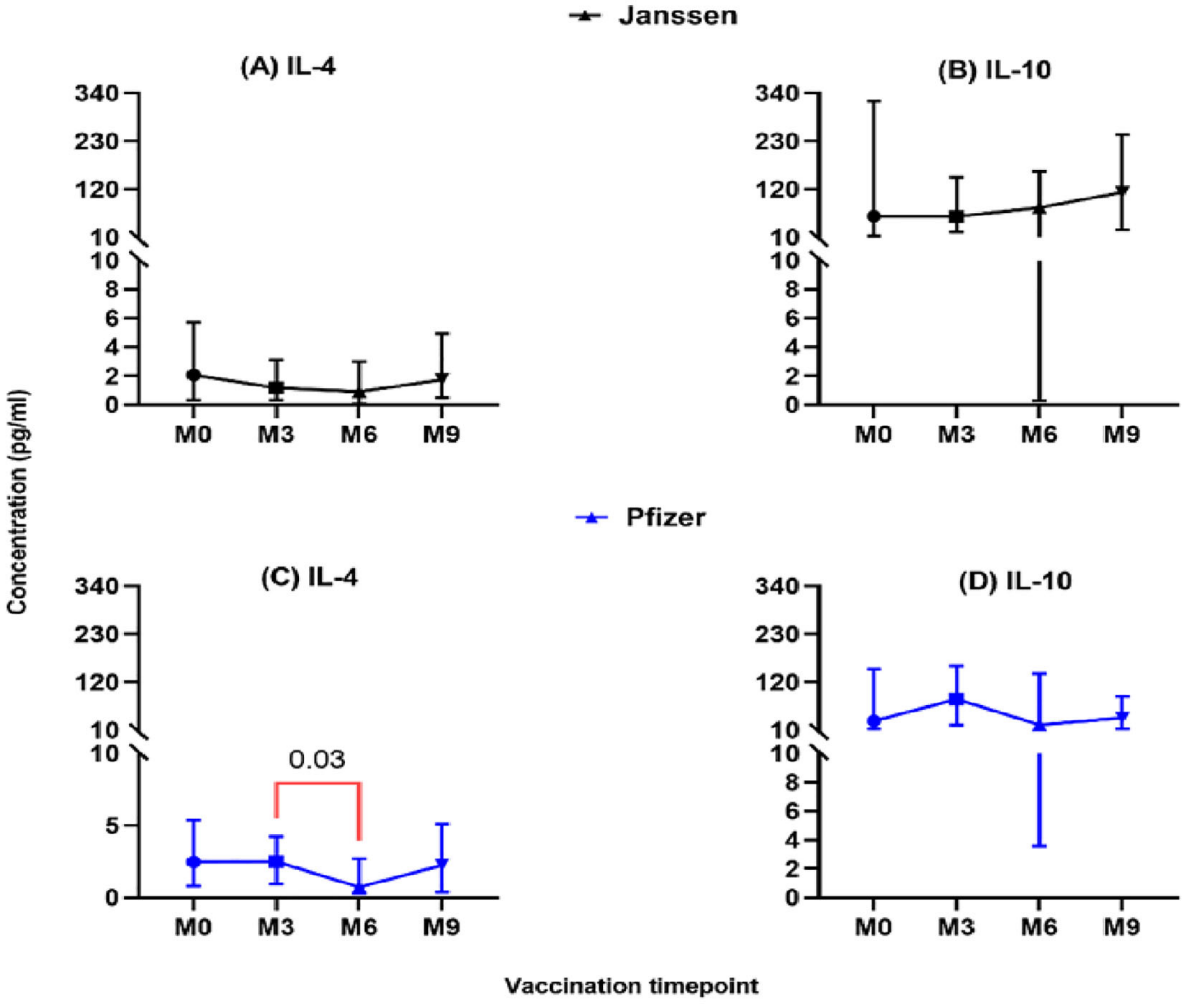
Longitudinal analysis of IL-4 and IL-10 responses (pg/ml) in individuals who received the Janssen **(A, B)** or Pfizer **(C, D)** booster vaccines. Friedman’s test, and Dunn’s multiple comparisons were used for across time point comparisons in the Janssen and Pfizer recipients. M0 = Samples taken before booster administration, M3, M6 & M9 = Samples taken at months 3, 6, and 9 post booster administration. Data points represent the median and the error bars, the minimum to maximum range.

The level of secreted T-helper 2 cytokine IL-4 was lower in both vaccine groups and did not differ significantly pre- and post-booster vaccination (M0 vs M3, M6 & M9; **Figure 3**). However, in the Pfizer group, IL-4 levels dropped significantly from (2.5pg/ml [0.95-5.1]) at month 3 to (0.73pg/ml [0.07-2.9]; p=0.03, **Figure 3C**) at month 6. Immunoregulation is key to ensuring optimal cytokine secretion and avoiding possible immunopathology. We assessed the immunoregulatory activity of the vaccines by measuring IL-10 levels. Appreciable levels were recorded in both vaccine groups pre- and post-booster vaccination but were not statistically significant (**Figures 3B, D**).

### Janssen and Pfizer booster vaccinated individuals exhibited similar magnitude of T-cell cytokine expression over nine months

**Figure 4:**
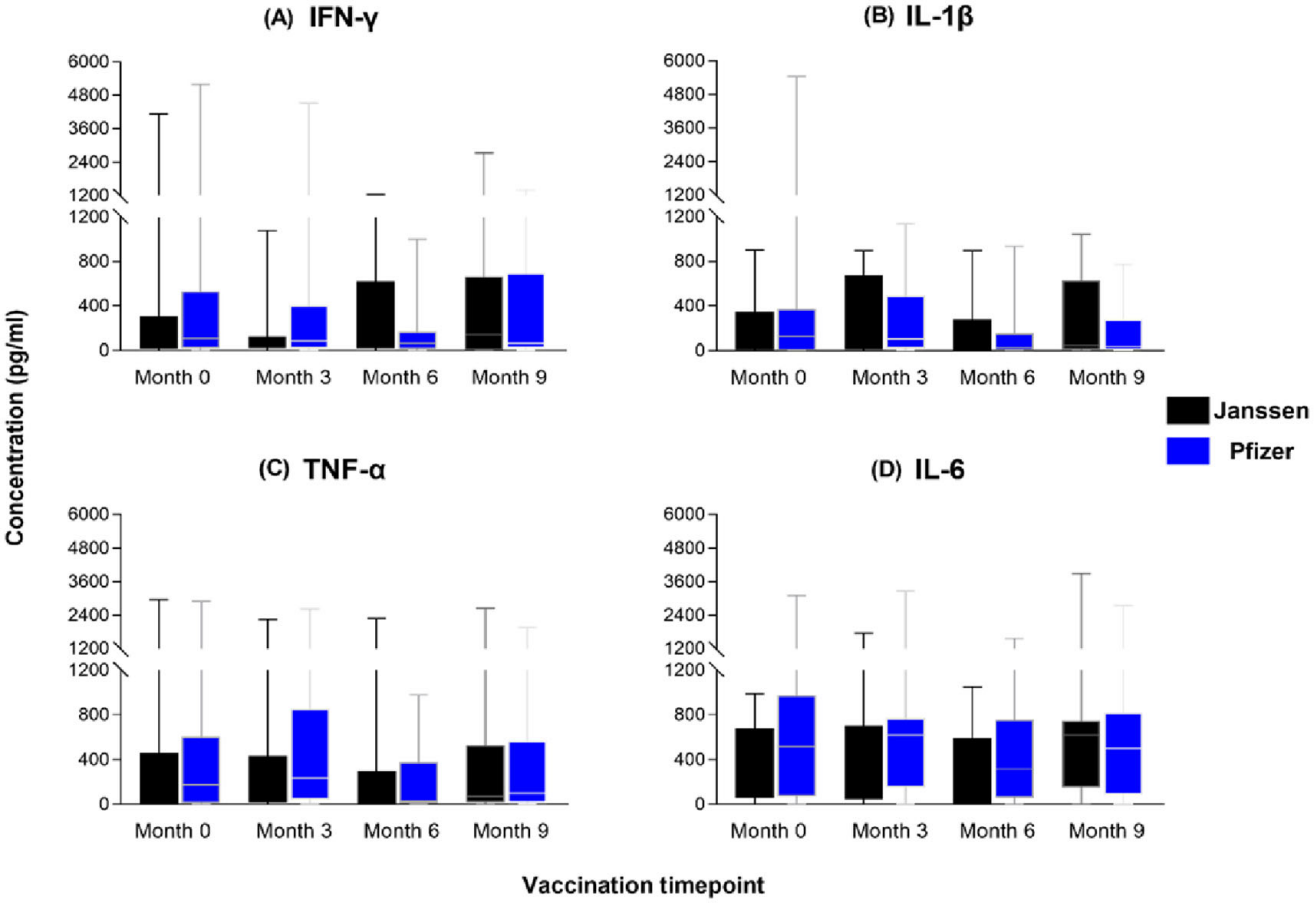
Magnitude of IFN-γ **(A)**, IL-1β **(B)**, TNF-α **(C)** and IL-6 **(D)** responses between Janssen and Pfizer booster vaccinated individuals. The data are represented as box and whisker plots; p-values were calculated at each timepoint using the Mann-Whitney U-test between the Janssen and Pfizer groups with no significant differences reported (p>0.05). Month 0 = Samples taken before booster administration, Month 3, 6 & 9 = Samples taken at months 3, 6, and 9 post booster administration. White horizontal lines represent the median while the whisker represents the minimum to maximum range.

To assess the magnitude of the induced immune responses between the Janssen and Pfizer vaccine recipients, we compared responses between the two vaccines at each of the four sampling timepoints using the Mann-Whitney U-test. We observed substantial production of T helper 1 associated cytokines; IL-1β, IFN-γ, TNF-α, and IL-6 (**Figure 4**) by both vaccines with no significant differences reported. Both vaccines also induced low levels of IL-2, IL-4, IL-12 and IL-17A across the study period (Figure not shown). IL-6 levels were the highest of the cytokines produced by both groups but were not statistically different between the two groups (p>0.05).

### Differences in cytokine expression between the Janssen and Pfizer booster vaccine groups

To assess cytokine expression differences between the Janssen and Pfizer booster vaccine groups over the 9-month study period, we fitted a Generalized Linear Mixed Models (GLMMs) for each cytokine. Our analysis revealed a lack of significant differences in cytokine levels between the two vaccine platforms.

As shown in **Table 2**, the Janssen vs Pfizer booster vaccine row for each cytokine represents the estimated coefficient of the difference in log-odds between the two groups. Our analysis found that the log-odds of having high IL-1β, IL-2, IL-4, IL-10, IFN-γ and TNF-α are greater in the Janssen booster recipients, however these were not significant (p >0.05; **Table 2**) indicating that there was no true difference between the effect of the booster vaccine type on the cytokine level.

**Table 2:** Summary of generalized linear mixed effects model for cytokine levels.

Fixed effect	Estimated coefficient	Std Error	z-value	p(>|z|)	AUC
GLMMs IL-1β					0.89
intercept	-1.00614	0.90293	-1.114	0.2651	
Janssen vs Pfizer booster vaccine	0.41558	0.84789	0.49	0.624	
GLMMs IL-2					0.98
intercept	-9.68431	3.62688	-2.67	0.00758	
Janssen vs Pfizer booster vaccine	0.87709	2.96565	0.296	0.76742	
GLMMs IL-4					0.99
intercept	-9.34342	4.36812	-2.139	0.0324	
Janssen vs Pfizer booster vaccine	0.06314	3.76948	0.017	0.9866	
GLMMs IL-6					0.9
intercept	-4.03839	1.65527	-2.44	0.0147	
Janssen vs Pfizer booster vaccine	-0.17211	1.40046	-0.123	0.9022	
GLMMs IL-10					0.98
intercept	-8.67E+00	5.02E+00	-1.727	0.0842	
Janssen vs Pfizer booster vaccine	1.22E+00	3.50E+00	0.348	0.7276	
GLMMs IL-12					0.99
intercept	-2.96E+01	6.36E+05	0	1	
Janssen vs Pfizer booster vaccine	-2.17E+01	2.05E+06	0	1	
GLMMs IL-17					0.98
intercept	-8.05968	4.12107	-1.956	0.0505	
Janssen vs Pfizer booster vaccine	-17.96874	2048.01312	-0.009	0.993	
GLMMs IFN-γ					0.69
intercept	-4.04811	1.59576	-2.537	0.0112	
Janssen vs Pfizer booster vaccine	1.20331	1.2731	0.945	0.3446	
GLMMs TNF-α					0.97
intercept	-6.98668	4.05077	-1.725	0.0846	
Janssen vs Pfizer booster vaccine	2.11251	2.14336	0.986	0.3243	

Odds of high cytokine levels (pg/ml) by Janssen booster vaccine compared to Pfizer booster vaccine, adjusting for confounders (Age, sex and Prior vaccine type).

### Longitudinal cytokine analysis of homologous and heterologous booster vaccinated individuals shows similar trends

To elucidate the effects of homologous (individuals receiving vaccines constructed on the same platform for primary and booster vaccinations) and heterologous (individuals receiving vaccines developed on different platforms for primary and booster vaccinations) vaccination on the durability of T-cell responses, the levels of secreted cytokines were compared across the nine months.

**Figure 5:**
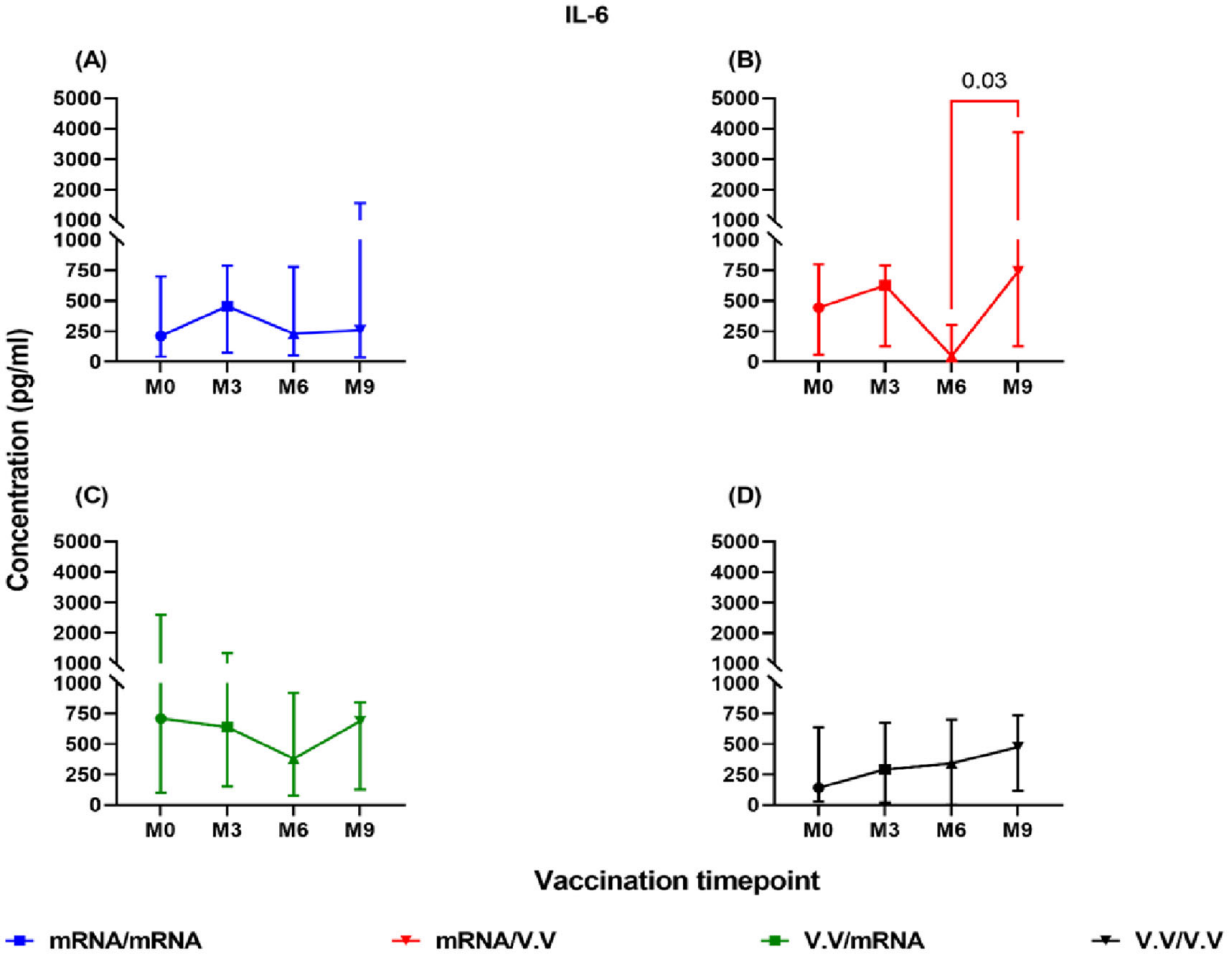
Levels of IL-6 (pg/ml) elicited by homologous and heterologous booster vaccinated individuals at Month 0 **(A)**, Month 3 **(B)**, Month 6 **(C)** and Month 9 **(D)** following Spike-RBD stimulation. Friedman’s test and Dunn’s multiple tests were used for comparisons Month 0 denotes samples taken before booster shot administration. M3, M6, and M9 denote samples taken at months 3, 6, and 9 post-booster vaccine administration, respectively. V.V (Viral vector recipients), RNA (mRNA recipients). Data points represent the median and the error bars, the minimum to maximum range. mRNA/mRNA (A, n=12), mRNA/V.V (B, n=6), V.V/V.V (C, n=18), V.V/mRNA (D, n=12).

Both groups of individuals who completed homologous mRNA as well as homologous viral vector vaccination exhibited appreciable levels of IL-6, TNF-α, IL-10, IL-1β, IFN-γ post-booster vaccination with no significant differences observed between time points (data not shown; p>0.05). There were also no timepoint differences in cytokines for the heterologous viral vector/mRNA recipients (p>0.05). However, IL-6 levels increased significantly in the heterologous mRNA/viral vector recipients at month 9 (median: 741 pg/ml) compared to month 6 (median: 46 pg/ml; p=0.03; **Figure 5**).

### Levels of some cytokines were higher at month 9 in the heterologous mRNA/viral vector group

**Figure 6:**
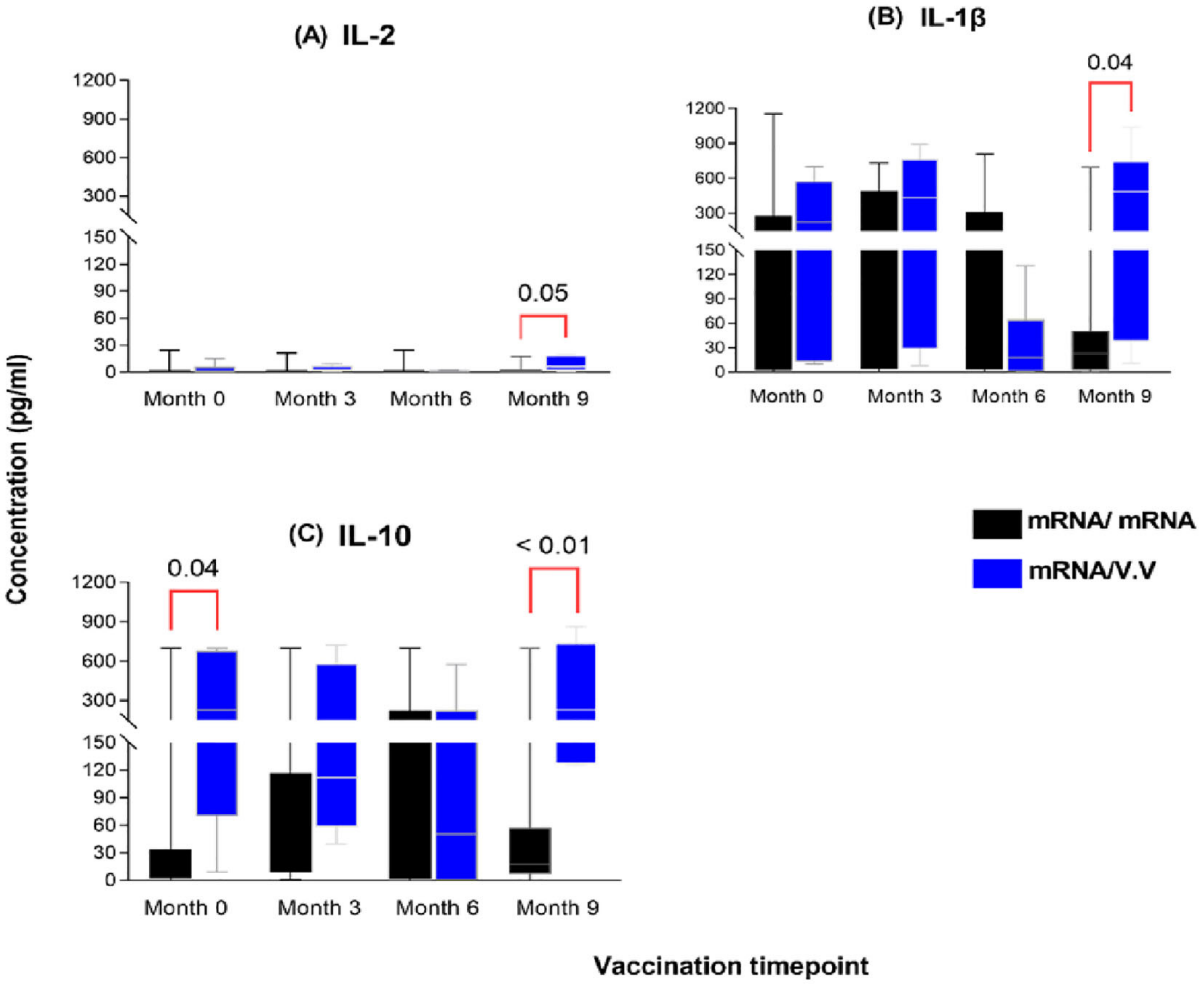
Magnitude of IL-2 **(A)**, IL-1β **(B)** and IL-10 **(C)** responses between individuals who received a primary mRNA vaccination and either an mRNA or viral vector booster vaccine. The data are represented as box and whisker plots; p-values were calculated at each timepoint between the mRNA booster and viral vector booster groups using the Mann-Whitney U-test. Month 0 = Samples taken before booster administration, M3, M6 & M9 = Samples taken at months 3, 6, and 9 post booster administration. Data points represent the median and the error bars, the minimum to maximum range.

To further assess which booster vaccination combination elicited a more efficient or robust cellular response, we compared the cytokine responses between persons who received the mRNA as a booster and those who received a viral vector booster vaccine at each timepoint. Nine (9) months post-booster administration, significantly higher levels of IL-2, IL-1β and IL-10 were observed in the viral vector group (median: 6.0 pg/ml; 484 pg/ml; 231 pg/ml) compared to the mRNA booster group (median: 0.78 pg/ml; 23 pg/ml; 18 pg/ml) (p-value: 0.05; 0.04; <0.01, **Figure 6**). There were no significant differences between the comparator groups for the remaining cytokines assessed.

### High T-cell activity confirmed by the levels of T-cell inhibitory molecules expression

To assess the kinetics of the T-cell activity within the study period, CD4+ and CD8+ T cells were analyzed for their immune checkpoint protein (PD-1, TIM-3 & CTLA-4) expression using flow cytometry (see **Supplementary Figure 1** for gating strategy used).

While low levels of inhibitory immune checkpoint proteins were observed in both Janssen and Pfizer booster recipients, no statistically significant differences in their expression were observed across pre- and post-booster timepoints (**Table 3**).

**Table 3:** Expression patterns of immune markers over nine-month study period.

Cells *(Median [IQR])*	Month 0	Month 3	Month 6	Month 9	p-value
Janssen					
CD4+	62 (47–68)	57 (47-65)	57 (41-68)	61 (48-70)	0.53
CD4+TIM-3+	2.6 (0.9-4.8)	2.9 (0.78-4.70)	1.9 (0.63-15)	3.3 (0.99-6.0)	0.3
CD4+CTLA-4+	2 (1.3-4.1)	3 (1.2-4.0)	3.3 (1.8-5.1)	2.2 (1.5-3.4)	0.48
CD4+PD-1+	5.6 (2.2-8.0)	4.9 (3.8-7.1)	4 (1.9-6.5)	4.5 (2.8-8.8)	0.18
CD8+	29 (24-41)	34 (26-42)	34 (23-41)	28 (22-38)	0.07
CD8+TIM-3+	5.6 (1.7-6.7)	3.5 (1.5-8.6)	2.1 (0.93-7.4)	3.8 (0.99-13)	0.2
CD8+CTLA-4+	2 (0.95-4.0)	1.9 (1.5-3.8)	2.1 (0.43-3.1)	1.7 (0.76-3.0)	0.16
CD8+PD-1+	4.1 (2.8-6.5)	4.5 (2.0-6.1)	5 (2.2-6.6)	3.8 (2.4-6.8)	0.41
Pfizer					
CD4+	60 (56-68)	57 (47-68)	59 (55-69)	65 (57-67)	0.12
CD4+TIM-3+	1.1 (0.74-3.9)	0.95 (0.43-2.9)	1.5 (0.86-3.7)	1.7 (0.77-3.2)	0.66
CD4+CTLA-4+	1.8 (1.2-2.7)	1.5 (0.97-2.3)	2.3 (1.5-3.1)	1.9 (1.5-3.3)	0.44
CD4+PD-1+	5.4 (4.0-6.4)	5.3 (2.7-6.0)	5.1 (3.4-8.1)	4.7 (3.6-5.3)	0.77
CD8+	27 (25-36)	32 (24-39)	30 (24-37)	27 (22-34)	0.9
CD8+TIM-3+	3.5 (1.6-7.3)	3 (1.2-8.5)	3 (0.98-7.1)	3.3 (2.1-5.5)	0.8
CD8+CTLA-4+	1.8 (0.91-4.8)	2 (1.2-5.6)	1.6 (1.2-4.4)	2.2 (1.1-4.3)	0.5
CD8+PD-1+	4.8 (2.7-7.5)	4.3 (2.5-11)	5.2 (3.3-7.6)	4.9 (3.5-11)	0.98

Median cell frequencies were compared across time points using Friedman’s test and Dunn’s multiple comparisons. P ≤ 0.05 was considered statistically significant. IQR: Interquartile range; TIM-3: T-cell immunoglobulin domain and mucin domain-containing protein 3; PD-1: programmed cell death 1; CTLA-4: Cytotoxic T-lymphocyte associated protein 4.

## Discussion

This study investigated the cellular immune responses and immune checkpoint molecule expression kinetics over a 9-month period in Ghanaian adults who received either the Pfizer (mRNA) or Janssen (adenoviral vector) vaccine as a booster. Our findings suggest that both vaccine platforms induced sustained cytokine responses for at least nine months, with a clear Th1-skewed immune profile. This Th1 bias was marked by higher levels of IL-1β, IFN-γ, TNF-α, and IL-6 relative to Th2, Th17, and Treg cytokines. This is consistent with previous research highlighting the significant role of Th1 cytokines in post-vaccination immunity (14, 27). The observed durability of the immune response likely stems from the unique mechanisms of the two vaccine platforms. Adenoviral vectors, such as Janssen’s, promote long-term antigen exposure in lymphoid tissues, which can lead to a long-lived population of effector and memory Th1 cells. This “memory inflation” phenomenon may account for the durable cellular immune responses observed in our study and others (28). In contrast, mRNA vaccines, like Pfizer’s, deliver transient instructions for Spike protein expression. While this induces a strong and rapid initial response, the transient nature of mRNA and its rapid degradation likely contributes to the early peak and subsequent decline in some cytokine levels (29). These differential mechanisms were reflected in the distinct temporal dynamics of key cytokines. The most highly expressed cytokine, IL-6, which is essential for B-cell differentiation, antibody synthesis, and T-cell regulation (30, 31), showed notable differences between the groups. In the Janssen group, IL-6 levels remained stable for the first six months and then increased significantly by month nine. In contrast, the Pfizer group exhibited an initial peak at three months, followed by a gradual decline. Similarly, TNF-α and IL-1β levels declined significantly over time in Pfizer recipients (**Figures 1G, H**), further highlighting the differential cytokine kinetics. These variations may reflect differences in the sustained antibody responses elicited by each vaccine given that all three cytokines are relevant for B cell activation, antibody production and enhancement of the innate immunity against respiratory infections (32, 33).

IL-2 supports T-cell activation, while IL-12 enhances Th1 responses and cell-mediated immunity, both crucial for effective vaccine responses to the related SARS-CoV (34, 35). However, in our study, we observed low levels of these cytokines in both vaccine groups at all timepoints, highlighting an insignificant role of these cytokines in generating the observed cellular immune response among study participants.

The controlled expression of IL-17 is likely beneficial, as excessive levels are linked to inflammatory pathology (36). We also observed differences in IL-4 kinetics: levels in the Pfizer group peaked at three months and then significantly declined by month six, while levels were sustained over nine months in the Janssen group. This finding strengthens the argument for plausible sustained antibody production in the Janssen group compared to the Pfizer group. IL-4 is known to activate and sustain humoral immune responses, including B-cell class-switching and antibody production (37). The overall low levels of both IL-4 and IL-17 relative to Th1 cytokines underscore the Th1-biased immune profile induced by both vaccines, consistent with a previous report by Salleh et al. (38).

The immunoregulatory cytokine IL-10 remained at appreciable levels throughout the nine-month period in both groups. This persistent expression is critical for modulating immune responses, preventing excessive inflammation, and reducing the risk of cytokine storms. As a regulatory cytokine, IL-10 also helps maintain long-term functional T-cell memory and immune homeostasis (39).

Following comparison of the magnitude of the induced cytokine responses between the Pfizer and Janssen vaccine groups, there was no significant difference across the nine months (**Figure 4**). This suggests that the two platforms induce comparable levels of cell-mediated immunity over time among vaccine recipients. This finding is asserted by the lack of significant difference in the log-odd effect of booster type on cytokine responses as observed by our GLMM. However, contrary to our findings, other studies reported a more robust immune response in mRNA vaccine recipients compared to adenoviral vaccine recipients, although these studies undertook these measurements for shorter periods up to six months and reported a decline in the said responses by the 6 month (40, 41). Also, a comparison of the booster dose’s response at Month 3 to the pre-booster response (Month 0) showed no significant change in cytokine levels although a 1.2-fold increase was observed. This fold-increase was similar to the 1.5-fold increase observed in serum by Alghamdi et al. (42).

A longitudinal analysis of individuals who received a viral vector booster after a primary mRNA regimen (heterologous) revealed a significant rise in IL-6 levels at six months post-booster, a pattern not seen in homologous (mRNA) recipients. The heterologous group also exhibited significantly higher expression of IL-2, IL-1β, and IL-10 nine months post-booster compared to the homologous group, suggesting a better Th1 and T-regulatory response. This finding aligns with emerging evidence that heterologous booster regimens can augment immune responses and potentially broaden protection compared to homologous regimens (43–45).

We also assessed the expression of immune checkpoint molecules such as CTLA-4, PD-1, and TIM-3, which are critical for regulating T-cell responses (46). High expression of these proteins can lead to T-cell “exhaustion” and diminished function (47). Importantly, we observed low expression of these molecules in both groups at all time points, similar to levels found in healthy individuals and below those observed in COVID-19 patients (>40% of CD4+ and CD8+ T-cells) by Diao et al. (48).This indicates a normal T-cell activation status and suggests that the booster dose did not induce exhaustion, allowing the T-cell population to remain functional and responsive. We also observed no significant boost in the cellular immune response post-booster compared to pre-booster levels, similar to findings of Busa et al. (49).

## Conclusions

The administration of an additional Pfizer (BNT162b2) or Janssen (Ad.26.COV2.S) booster dose resulted in sustained Th1-biased cellular responses that could be clinically protective. Across the study timepoints, levels of some cytokines were more sustained in the Janssen group compared to the Pfizer group. However, timepoint-specific comparison between the two booster vaccinated groups did not show any significant differences in the cytokine expression levels. Heterologous vaccination regimen showed trends toward enhanced durable cellular immune responses compared to the homologous group. Recipients of both booster vaccine types showed durable and comparable T-cell response with no signs of T-cell exhaustion or excessive inflammation, highlighting the safety of these vaccines. These findings contribute to understanding long-term cellular immunity following booster vaccination and underscore the importance of monitoring cytokine dynamics. Moreover, these results provide important suggestions for improving COVID-19 booster immunization strategies among African populations, particularly under heterogeneities of vaccine availability and platform use in various settings.

## Data Availability

The original contributions presented in the study are included in the article/**Supplementary Material**, further inquiries can be directed to the corresponding author/s.
