# Supplementary material for: Longitudinal evaluation of T-cell responses to Pfizer-BioNTech and Janssen SARS-CoV-2 vaccines as boosters in Ghanaian adults
Source: Front Immunol. 2025 Sep 12;16:1643083. doi: 10.3389/fimmu.2025.1643083 (PMC12463971; doi:10.3389/fimmu.2025.1643083)
Supplement: Supplementary file 1 [file Supplementaryfile1.docx]

Supplementary Material


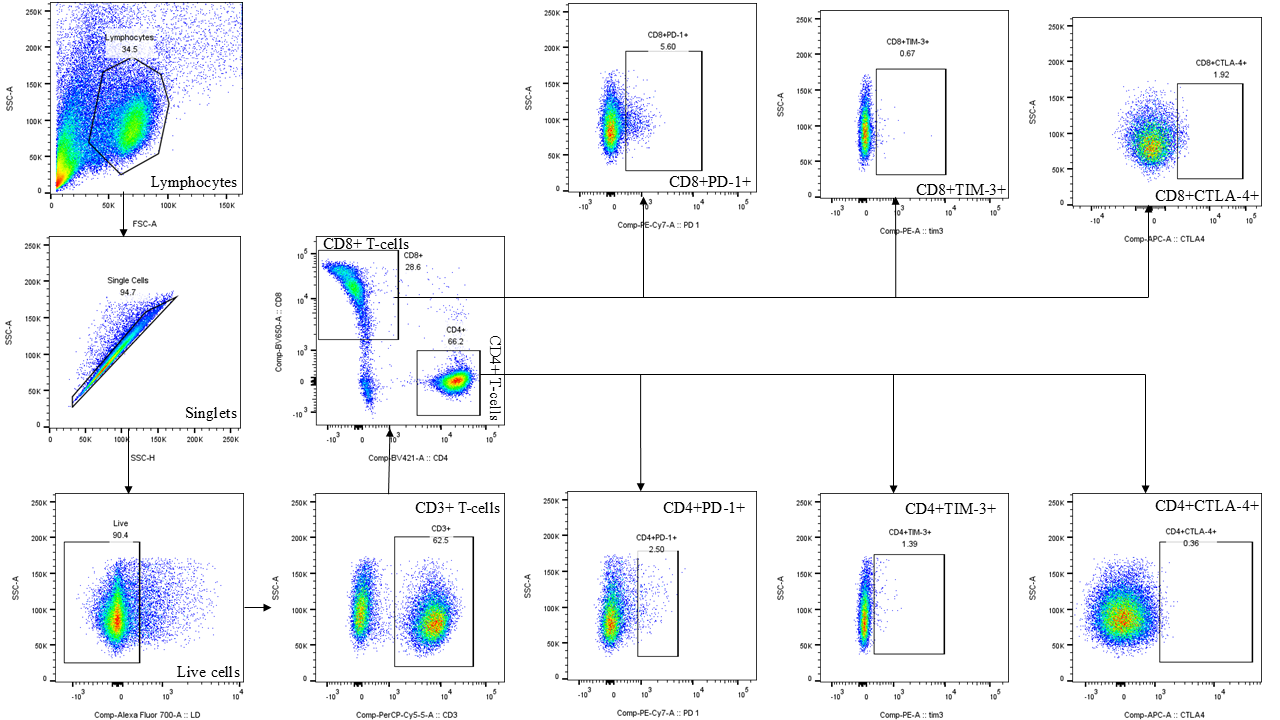


**Supplementary Figure 1: Representative flow cytometry plot indicating manual gating strategy for the analysis of immune checkpoint molecules (PD-1, TIM-3, and CTLA-4) on CD4+ and CD8+ T-cells.** A gating strategy identifying lymphocytes, singlets, live cells, CD3+ T-cells, CD4+, CD4+PD-1+, CD4+CTLA-4+, CD4+TIM-3+, CD8+, CD8+PD-1+, CD8+CTLA-4+, and CD8+TIM-3+.
